# Enterocutaneous Fistulas: Current Management

**DOI:** 10.3390/nu18121926

**Published:** 2026-06-14

**Authors:** Amier Mohamed Rashed, April Mendoza, D. Dante Yeh

**Affiliations:** 1 Department of Surgery, University of Miami Miller School of Medicine, Miami, FL 33136, USA; 2 Department of Surgery, University of California, San Francisco (UCSF)—East Bay, Oakland, CA 94602, USA; apmendoza@alamedahealthsystem.org; 3 Denver Health Medical Center, University of Colorado, Denver, CO 80204, USA; dante.yeh@dhha.org

**Keywords:** enterocutaneous fistula, enteroatmospheric fistula, fistula, short bowel syndrome, intestinal failure, total parenteral nutrition, enteral nutrition

## Abstract

**Background:** Enterocutaneous fistulas (ECFs) and enteroatmospheric fistulas (EAFs) are rare but highly morbid complications that most commonly arise after abdominal surgery. Outcomes have improved with advances in multidisciplinary care and with increasing research on how to best manage them; however, they remain associated with significant morbidity, high mortality, and prolonged hospitalization. Optimal timing of definitive repair is unknown, with many high-volume centers waiting 6–12 months, though emerging data suggest that earlier intervention may be feasible in carefully selected patients. Given their complexity and variability in management, a comprehensive review of current evidence is needed. **Methods:** A narrative review of the literature was conducted with emphasis on the classification, pathophysiology, and multidisciplinary management of ECFs and EAFs. Relevant studies addressing fluid and sepsis control, nutritional optimization, wound care, pharmacologic therapies, and interventional strategies were reviewed. **Results:** The management of ECFs requires a staged approach focused on fluid resuscitation, sepsis control, wound management, and nutritional optimization. Spontaneous closure can occur, and is most commonly within the first two months. Nutritional optimization through enteral and/or parenteral nutrition or fistuloclysis plays a vital role in improving outcomes. Therapies such as negative pressure wound therapy, biologics, and pharmacologic agents may support spontaneous closure and fistula control. In non-healing fistulas, surgical repair remains necessary, with optimal time for surgery at least 6–12 months from fistula development. **Conclusions:** ECFs and EAFs remain complex surgical challenges. Outcomes have improved due to advances in nutritional support and wound management, and the emergence of minimally invasive techniques. Standardization of treatment protocols and further research into novel therapy may further enhance outcomes and limit variability in management.

## 1. Introduction

An enterocutaneous fistula (ECF) is defined as an aberrant communication between the small or large bowel and the skin of the abdomen and is a devastating complication occurring after approximately 1% of emergency and elective laparotomy operations [1]. An enteroatmospheric fistula (EAF) is a subset of ECFs where the communication is between the bowel and the atmosphere without any surrounding skin or soft tissue [2]. ECFs are associated with considerable morbidity, such as increased ICU and hospital length of stay (LOS), hospital stays, and hospital charges [1,3,4]. Infectious complications are generally the rule, occurring in over half of all ECF/EAF patients [5]. Many patients develop persistent inflammation–immunosuppression catabolism syndrome (PICS) and the associated prolonged requirement for organ support [6]. In addition to their surgical complexity, ECFs frequently result in significant nutrient, fluid, and electrolyte losses and are among the most common causes of type 2 intestinal failure requiring prolonged enteral and/or parenteral nutrition support. Historically, the mortality rate associated with ECFs was >20% [7] and deaths were mainly attributed to sepsis, malnutrition, and fluid/electrolyte disturbances [8]. However, due to advancements in nutrition support, percutaneous interventional radiology techniques, and overall medical progress, modern series routinely report mortality rates < 10% [5,9,10]. Not surprisingly, ECF patients report poor quality of life [11].

ECFs and EAFs represent some of the most complex forms of acquired intestinal failure encountered in practice. Beyond the immediate risks of sepsis, fluid loss, and wound complications, these patients frequently develop profound malnutrition, micronutrient deficiencies, impaired intestinal absorption, and prolonged dependence on specialized nutrition support. Contemporary management therefore extends beyond surgical decision-making and requires a multidisciplinary strategy focused on intestinal rehabilitation, optimization of nutritional status, preservation of gut function, and restoration of functional independence. Because ECFs and EAFs are relatively uncommon and highly heterogeneous, management practices vary considerably between institutions and providers. In this narrative review, we summarize current evidence regarding multidisciplinary management of ECFs and EAFs, with particular emphasis on nutritional assessment, enteral and parenteral nutrition strategies, fistuloclysis, chyme reinfusion, intestinal rehabilitation, and the integration of nutritional optimization with wound care, source control, and definitive surgical reconstruction.

This manuscript was conducted as a narrative review. A literature search was performed using PubMed/MEDLINE, Embase, and Google Scholar from inception through January 2026. Search terms included “enterocutaneous fistula,” “enteroatmospheric fistula,” “intestinal failure,” “fistuloclysis,” “chyme reinfusion,” “parenteral nutrition,” “enteral nutrition,” “negative pressure wound therapy,” and related terms. Priority was given to clinical guidelines, systematic reviews, meta-analyses, prospective studies, and large retrospective series. Additional references were identified through manual review of bibliographies and expert knowledge of the field. This narrative review was prepared in accordance with the Scale for the Assessment of Narrative Review Articles (SANRA) recommendations.

### Classification

Multiple systems can be used to classify and describe ECFs. Daily volume of output is one of the primary and most important methods used for classification. Based on output, ECFs are classified into: high output (>500 mL/24 h), moderate output (200–500 mL/24 h), and low output (<200 mL/24 h) [8]. ECFs may also be classified according to etiology. The vast majority of ECFs occur after surgery due to anastomotic leaks or unrecognized enterotomies, with the remaining occurring due to inflammatory bowel disease, malignancy, or radiation enteritis [12]. Classifying ECFs based on their site of origin in the GI tract is also common. Proximal fistulas are those arising from the stomach, duodenum, jejunum, or proximal ileum while distal fistulas are those that arise from the distal ileum or colon [13].

ECFs represent a form of intestinal failure (IF). The European Society for Clinical Nutrition and Metabolism (ESPEN) defines IF as the reduction of intestinal function below the minimum necessary for the absorption of macronutrients and/or water and electrolytes, such that intravenous (IV) supplementation is required to maintain health and/or growth [14]. IF can be further classified into three types. Type 1 IF is defined as acute, short-term, and usually self-limiting. Type 2 IF is defined as a prolonged acute condition, often in metabolically unstable patients, requiring complex multidisciplinary care and IV supplementation over a period of weeks or months. Type 3 IF is a chronic condition, in metabolically stable patients, requiring IV supplementation over months or years and may be reversible or irreversible [14]. Patients with ECFs usually fit into the criteria established in types 2 and 3 IF.

While the rate of spontaneous fistula closure is variable in the literature, most studies demonstrate a spontaneous closure rate of 20–37% [7,15,16,17,18,19]. Spontaneous closure most commonly occurs in the first two months after fistula development, with 90% of closures occurring in the first month. Spontaneous closure is less likely (though not impossible) after the second month [20,21,22,23]. Factors that favor spontaneous closure include: surgical etiology, no distal obstruction, fistula length > 2 cm, low-output fistulas, fistulas associated with appendicitis and diverticulitis, no ongoing infections, balanced electrolytes, and care by a tertiary care center. Inflammatory bowel disease (IBD), radiation, malignancy, and proximal fistulas especially if involving the duodenum or jejunum portend a decreased likelihood for spontaneous closure [15,17,24,25,26], while exposed mucosa signals that the fistula will never spontaneously close.

Although spontaneous closure is more likely for low-output fistulas, colonic fistulas, and ECFs resulting from complications of surgery (vs. IBD), [17] it is possible for high-output ECFs to spontaneously close [27]. For fistulas that do not spontaneously close and require operative repair, reported ECF recurrence rates range from 8 to 20% [9,10,15,18,28,29,30,31,32].

## 2. Principles of Management

**Figure 1:**
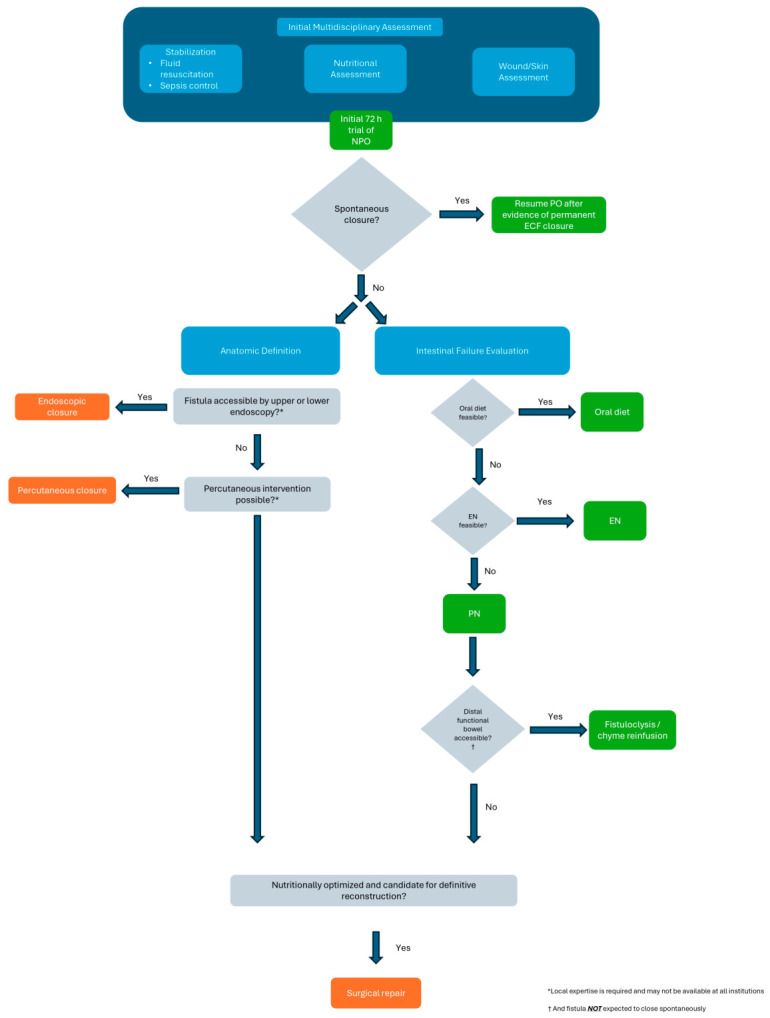
Nutritional rehabilitation pathway for patients with enterocutaneous and enteroatmospheric fistulas. * Local expertise is required and may not be available at all institutions; † And fistula NOT expected to close spontaneously.

The principles of management of ECFs include: (1) initial stabilization and fluid resuscitation, (2) control of sepsis, (3) management of the surgical wound and protection of the nearby skin, (4) anatomic definition, and (5) adequate nutrition provision and correction of electrolyte abnormalities. If these measures do not result in spontaneous closure, then the final stage is proceeding with the definitive operative intervention. ECF patients benefit from early involvement of multidisciplinary teams that can provide complex nutrition support, wound care, advanced interventional endoscopic and radiologic procedures, and complex abdominal wall reconstruction [33,34]. Management of ECF patients at specialized centers with experienced providers and structured treatment pathways has been associated with improved outcomes [23,29,35,36]. The multidisciplinary treatment approach advocated in this review is summarized in Figure 1. Following stabilization and an initial period of bowel rest, patients undergo parallel anatomic definition and intestinal failure evaluation, which guide nutritional rehabilitation, consideration of minimally invasive therapies, and eventual candidacy for definitive reconstruction. Nutritional optimization remains a central component throughout this process.

### 2.1. Initial Stabilization and Fluid Resuscitation

It is critical to remain vigilant about the fluid status throughout the disease course. ECF patients can have extremely high fistula outputs, up to 4–6 L per day. Detailed and meticulous charting of fluid intake (by IV and enteral routes) and fistula and urine output is critical to adjusting fluid administration to maintain euvolemia. During the early period of stabilization, electrolytes should be measured daily and repleted accordingly. As the clinician becomes more familiar with the patient’s average daily fluid requirements, lab monitoring frequency can decrease. In the outpatient setting, electrolytes are usually monitored weekly in high-output fistula patients, and monitoring can be less frequent in moderate- and low-output patients.

In the authors’ experience, patients with newly diagnosed ECFs are initially managed with approximately 72 h of bowel rest. This allows assessment of baseline fistula output in the absence of gastrointestinal stimulation and facilitates fluid and electrolyte stabilization. Importantly, some patients experience a substantial reduction in fistula output and a subset may achieve spontaneous fistula closure without requiring additional interventions. Although the optimal duration of bowel rest has not been established and supporting evidence is limited, the authors have found this strategy useful in identifying patients who may respond to conservative management alone. If fistula output does not substantially improve during this initial period, oral intake is resumed and subsequent nutritional planning is individualized based on anatomy, absorptive capacity, and clinical status.

### 2.2. Sepsis and Source Control

The presence and persistence of sepsis will make recovery extremely unlikely. An undrained intraabdominal infection will keep the body in a catabolic state, rendering attempts at optimizing the patient’s nutritional status ineffective. Remaining vigilant for signs of infection and sepsis in patients with ECFs is necessary. Rapid deterioration is not unexpected in these patients due to their longstanding debilitation and often malnutrition.

Open surgical source control of abscess is not recommended and often impossible due to a “hostile abdomen”; therefore, percutaneous drainage is strongly recommended. However, percutaneous drainage may be difficult and inadequate. For example, the intraabdominal abscess will not have a safe “window” for an interventional radiologist (IR) to access without puncturing surrounding bowel. In selected cases and after multidisciplinary discussion, some centers may accept a transenteric drainage approach when no safe alternative route exists and source control is deemed essential. If the IR is unwilling to perform percutaneous drainage, then open surgical drainage will occasionally be necessary to obtain adequate source control. However, surgery should be considered a last resort in a clinically deteriorating patient. If attempted, the goal of surgical intervention at this time should only be source/sepsis control. Attempting to perform an anastomosis will inevitably lead to further anastomotic leakage and the need for additional future bowel resections. Exteriorization of the ECF or leaking anastomosis may be attempted, though in the authors’ experience, the bowel and mesentery are usually too inflamed and scarred to mobilize to the skin surface.

### 2.3. Skin and Wound Management

**Figure 2:**
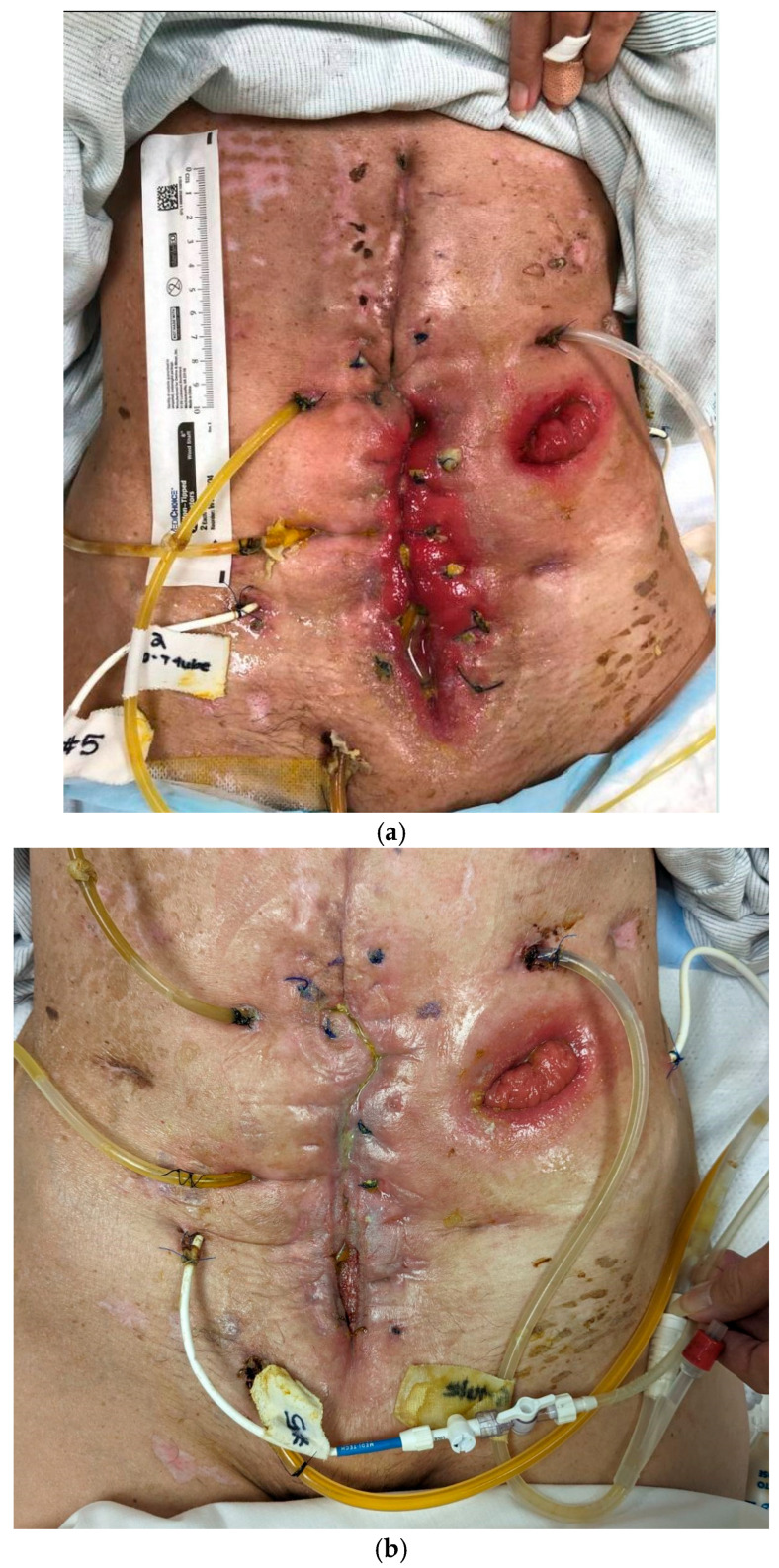
(**a**) Uncontrolled fistula causing severe skin irritation and pain. (**b**) Same patient after two weeks of intensive daily wound care.

Adequate skin care and wound management are imperative in the successful management of ECFs. Due to the presence of digestive enzymes in fistula effluent, skin excoriation begins within hours and, if left uncontrolled, can lead to severe pain, and skin and soft tissue infections (Figure 2). Additionally, patients with inadequately managed high-output fistulas often experience severe stress and become demoralized.

**Figure 3:**
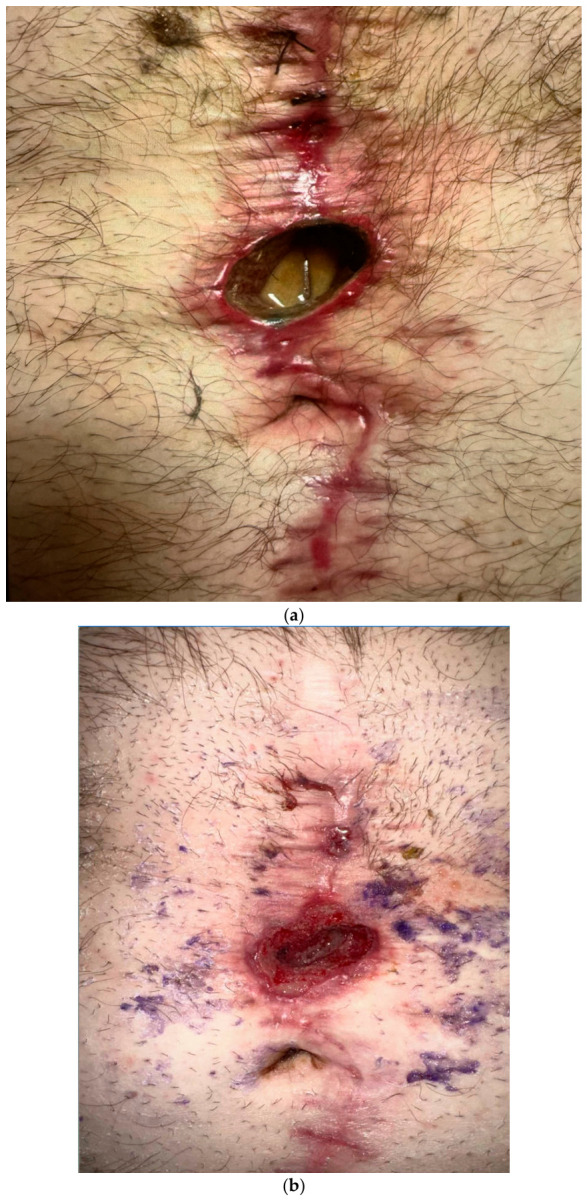

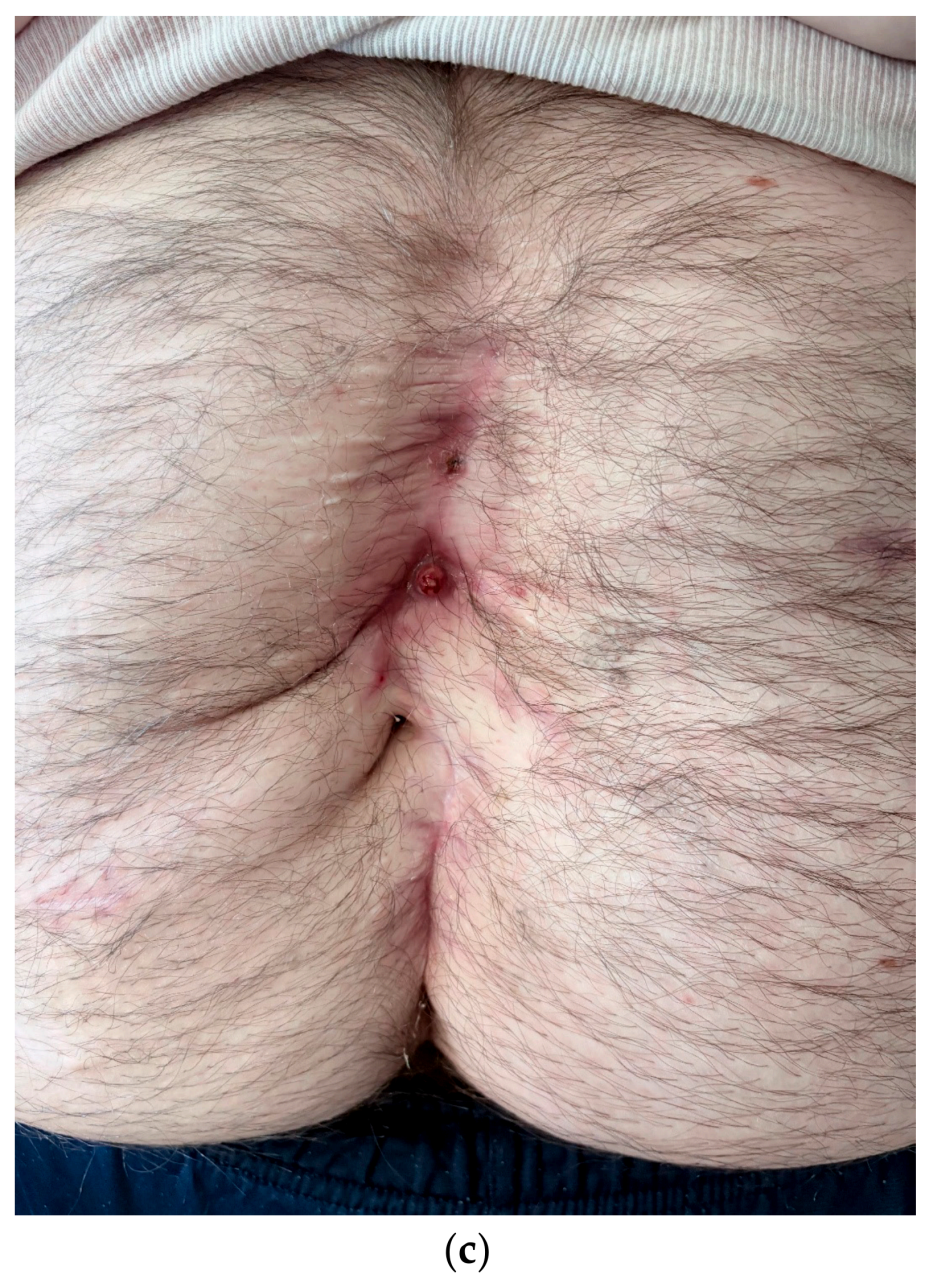
(**a**) Initial presentation of high-output enterocutaneous fistula. (**b**) Early wound evolution after initiation of negative pressure wound therapy (NPWT) with improving surrounding skin and progressive development of granulation tissue. (**c**) Complete fistula closure and restoration of skin integrity.

A variety of wound managers, stoma appliances, pastes, and dressings are available and can be used for patients with high fistula output. Ostomy specialists and wound care nurses are essential in guiding care and can provide recommendations tailored to individual patient needs. The use of negative pressure wound therapy (NPWT) is a relatively recent addition to the armamentarium for wound management. Early reports raised the concern of NPWT (or vacuum-assisted closure (VAC)) leading to the development of new ECFs [37,38]. However, after recognizing the risk of applying direct suction on the viscera and modifying the technique, multiple more recent studies have demonstrated that the use of NPWT can be highly beneficial in treating ECFs and EAFs, both in managing the wound and also for inducing spontaneous closure [39,40,41,42,43,44,45,46,47,48,49] (Figure 3).

## 3. Optimization of Nutrition Status

ECF/EAF patients are often malnourished at first encounter, especially considering that malnutrition is a recognized risk factor for anastomotic leaks. Even in well-nourished patients, periodic assessment of nutritional status is warranted for all ECF patients [50]. A nutrition-focused physical exam may be challenging due to total body fluid anasarca or edema [51]. Biomarkers and serum proteins have a complicated role in guiding nutrition plans for patients with ECFs. Serum proteins such as albumin and prealbumin (also known as transthyretin) were historically used to diagnose malnutrition and monitor nutritional recovery. However, it is now recognized that these proteins are negative acute phase reactants and are more reflective of inflammation rather than nutritional status. The American Society for Parenteral and Enteral Nutrition (ASPEN) position paper on this topic clearly recommends against using albumin and prealbumin as nutrition markers [52]. Hand grip strength (HGS) is an objective, inexpensive, and easily repeatable measurement that has been repeatedly validated as an accurate measure of overall physical functioning and nutrition [53]. In the authors’ practice, HGS measurements are obtained weekly in hospitalized patients and monthly in outpatients to monitor recovery and nutritional rehabilitation. Although no evidence-based threshold has been established specifically for ECF patients, the authors generally target recovery to at least 80% of the lower limit of age- and sex-adjusted normal values.

The ASPEN/FELANPE (Federación Latinoamericana de Terapia Nutricional, Nutrición Clínica y Metabolismo) clinical guidelines for the management of ECFs suggest that “oral diet or enteral nutrition (EN) may be feasible and tolerated in patients with low-output (<500 mL/d) ECFs” [54]. If well-tolerated and there is good control of ECF effluent, an oral diet has several advantages. While an oral diet can provide a patient with their required nutritional goals, it is also of psychological benefit, maintains a healthy gut microbiome, and maintains gut mucosal integrity [50]. While oral nutrition alone is generally preferred, a substantial proportion of ECF patients (especially high-output and proximal) will require supplemental enteral nutrition (EN) or parenteral nutrition (PN). Although not validated specifically in ECF patients, the authors occasionally perform an acetaminophen absorption test (AAT) as an adjunctive assessment of proximal gastrointestinal absorptive capacity. This approach is based on physiologic principles and extrapolation from prior studies evaluating gastric emptying and small intestinal absorption [55,56]. When 1000 to 1500 mg of acetaminophen is administered orally, the serum acetaminophen level drawn approximately 1 h later is approximately 10 mcg/mL in normal healthy volunteers. A very low or undetectable level can alert the clinician that proximal oral absorption may be compromised and that EN or PN may be required to maintain health and hydration. Additionally, upper gastrointestinal (GI) fluoroscopic anatomic mapping studies are obtained on all ECF patients after the initial stabilization period. In the authors’ practice, fluoroscopic mapping studies are reviewed not only for anatomy but also for transit time from the stomach to the fistula. Although this approach has not been formally validated, very rapid transit times (e.g., <30 min) may suggest limited absorptive opportunity and may prompt consideration of supplemental EN/PN.

In the authors’ practice, micronutrients are assessed at baseline and approximately every six months in ECF patients requiring long-term PN. Some experts recommend providing supplemental micronutrients, especially vitamin C (ascorbic acid), zinc, copper, and selenium, due to increased losses through fistula effluent [57]. This topic remains poorly researched, though oral/enteral supplementation is usually low-cost and the risk/benefit profile generally favors empiric supplementation.

### 3.1. Energy/Protein Prescription

Weight-based equations (such as 25 kcal/kg) or predictive equations (such as the Harris–Benedict equation) can be used to determine energy requirements. Indirect calorimetry is more accurate in determining energy needs as it can calculate the resting energy expenditure (REE), but is not widely available. The ASPEN/FELANPE clinical guidelines recommend protein at 1.5–2 g/kg/day for ECF management and up to 2.5 g/kg/day for those with EAFs [54]. Note that these equations only serve as a starting point and that frequent reassessment is necessary to ensure that the patient is responding to the prescription through restoration of lost weight (particularly lean body mass), improving strength and physical functioning, and ongoing healing of open wounds. If the patient has reached a plateau or is deteriorating, the clinician should not hesitate to increase energy and/or protein. Nitrogen balance studies may be obtained to help guide protein prescription, especially for patients receiving PN [58]. However, nitrogen balance may not be reliable if the clinician is unsure of how much oral/enteral intake is being absorbed. Although optimal nitrogen balance targets in ECF patients remain uncertain, the authors generally aim for a positive balance of approximately +2 to +4 g/d nitrogen balance during active nutritional rehabilitation and approximately neutral balance once the patient has achieved their target weight and physical functioning status.

### 3.2. Enteral vs. Parenteral Nutrition

In cases where an oral diet is poorly tolerated or inadequate, supplemental EN (or oral nutritional supplements) should next be considered. Standard polymeric formulas are recommended as they have been associated with optimal intestinal rehabilitation. While elemental and semi-elemental formulas may, in theory, be more easily absorbed, they often are unpalatable (thus requiring long-term percutaneous enteral access for maximal compliance), are much more expensive, and have higher osmolarity which sometimes leads to higher fistula output. EN may be administered above the fistula, below the fistula (see fistuloclysis section below), or both.

In cases where EN is not well-tolerated (often due to pain, cramping, or excessively increased fistula output with malabsorption), then supplemental PN should be initiated [59]. Prior to the advent of PN in the 1960s, ECF patients had high mortality rates, mainly due to multiorgan failure and malnutrition. After the introduction of PN, though, strict bowel rest (nil per os, NPO) and total PN (TPN) became the standard of care for all ECF patients [22,60,61] and the mortality decreased to approximately 20% [7]. In recent decades, it was recognized that the complete cessation of bowel stimulation has negative effects on mucosal atrophy and cholestasis, contributing to intestinal failure-associated liver disease (IFALD), formerly known as parenteral nutrition-associated liver disease (PNALD) [62]. In modern practice, even for patients requiring 100% nutrition support by PN, ongoing oral or EN is encouraged for non-nutritive benefits [25]. Beyond the usual risks associated with PN in general (hyperglycemia, refeeding syndrome, and deep vein thrombosis), ECF patients on PN have higher rates of catheter-related bloodstream infections compared to non-ECF PN patients [63].

### 3.3. Fistuloclysis and Chyme Reinfusion

It is well-established that defunctionalized intestines experience muscular atrophy and loss of villi [64,65]. Studies in patients with temporary defunctionalizing ileostomies have demonstrated that preoperative stimulation with saline or ostomy effluent (chyme) results in improved postoperative outcomes such as earlier return of bowel function and shorter hospital length of stay [66,67,68,69]. Fistuloclysis (also known as “distal feeding”) refers to feeding EN formula to the distal intestine via the distal (efferent) limb of the fistula and offers substantial benefits [70,71,72,73,74]. Note that fistuloclysis is distinguished from “chyme reinfusion” (otherwise known as “reinfusion of succus entericus” [75], “extracorporeal stool transport” [76], or “continuous extracorporeal stool transport” [77]), which involves collecting the effluent from the fistula and “re-feeding” this content into the distal bowel [78,79]. Multiple studies have reported success in using fistuloclysis (with or without chyme reinfusion) to improve liver function tests, reduce intravenous hydration requirements, or wean patients off PN altogether [80,81,82,83,84,85]. In addition to providing nutrition and allowing for reabsorption of GI secretions such as bile, chyme reinfusion also decreases the volume of fistula output via a mechanism known as the “ileal brake” [86,87,88,89,90,91] and may also restore normal gut microbial biodiversity [92]. One study even reported lower rates of fistula recurrence after definitive surgery in patients receiving chyme reinfusion compared to those receiving standard care [93]. With increasing reports of clinical benefits [78,94,95], fistuloclysis should be strongly considered in patients who have significant bowel length distal to the ECF. However, it must be acknowledged that nearly 40% of patients will ultimately not tolerate chyme reinfusion, usually due to pain during chyme infusion [81]. Fistuloclysis requires complete knowledge of the gastrointestinal anatomy. The proximal and distal fistula sites should be clearly delineated and distal obstruction must be completely ruled out. In the event that distal anatomy is not accessible from the abdominal wall, percutaneous direct jejunostomy can be considered [96]. The ideal candidate for fistuloclysis is hemodynamically stable, with no active infections, and no possibility of spontaneous resolution of the fistula in the near future.

Overall, the benefits of fistuloclysis can include: liberation from PN, lower cost of treatment, improvement in electrolytes, reduction in risk of bacterial translocation, improved liver function, faster return of gastrointestinal function postoperatively, and shorter hospital length of stay [69,82,85]. Complications can include tube dislodgment, underfeeding, and leakage-related skin corrosion [85].

### 3.4. Pharmacological and Novel Agents in the Management of Intestinal Failure and ECFs

Anti-secretory and anti-motility agents are the mainstay of effluent control. Common anti-secretory agents include octreotide, H2-blockers, and proton pump inhibitors [97]. Common anti-motility agents, such as loperamide, diphenoxylate/atropine, and tincture of opium, mostly rely on the μ opioid receptor. Note that loperamide requires absorption and entero-hepatic circulation. In high-output fistulas, a significant proportion of an oral loperamide dose may be lost and therefore larger doses of loperamide are sometimes required to achieve an effect [97,98]. Other agents to be considered are bulking agents that can slow transit time, especially forms of soluble fiber. However, it should be noted that most dietary fiber supplements contain a mixture of soluble and insoluble fiber. Insoluble fiber acts as an osmotic agent and can increase small bowel fistula outputs. Additionally, agents that thicken intestinal content can make fistuloclysis difficult. Risks and benefits of each strategy should be weighed in every individual case.

The value of octreotide remains controversial [99,100,101,102,103]. Several studies [104] have demonstrated that while fistula output may decrease initially, the effects of octreotide are short-lived. This is most likely due to receptor adaptation and hormonal counter-regulation [101,105]. The mechanism of action of octreotide includes vasoconstriction (amongst other mechanisms) and therefore may be counterproductive to wound healing and intestinal rehabilitation [106,107,108]. However, there does exist some clinical evidence of octreotide helping to spontaneously close ECFs [100]. Given the conflicting literature, the authors reserve octreotide for selected patients as an adjunctive therapy when reducing fistula output or promoting closure, which may help avoid operative intervention.

Teduglutide, a glucagon-like peptide (GLP)-2 analogue, is an agent that has been proven to be effective for bowel rehabilitation in patients with short bowel syndrome (SBS) by promoting mucosal growth, reducing intestinal losses, and enhancing absorption [104,109]. Additionally, teduglutide can improve mesenteric blood flow and enhance intestinal epithelial barrier function [104,110,111]. A small randomized cross-over study demonstrated encouraging findings when teduglutide was added to standard care for the treatment of low-output ECFs [112]. However, this medication is expensive and is currently approved only for the treatment of SBS.

The use of stem cells to treat ECFs is an exciting potential future treatment under active investigation. The premise is to inject stem cells (adipose-derived, mesenchymal, and/or bone marrow-derived stem cells) locally into fistulas to promote closure. In a comprehensive systematic review, enhanced closure rates and improved short- and long-term outcomes were seen in patients who received stem cell therapy in a variety of fistula types which was not limited to ECFs or EAFs [113].

## 4. Non-Surgical and Surgical Interventions

### 4.1. Percutaneous Management of ECFs

Percutaneous drainage is frequently employed in the management of ECFs, especially for sepsis and source control [114,115]. There has been continued interest in the role of percutaneous therapies in the closure of fistulous tracts. A variety of materials have been reported in the literature including tissue sealants, gel foam, n-butyl cyanoacrylate (NCBA), [116] and decellularized porcine small intestinal submucosa [117]. Most of the data for percutaneous closures have been confined to case reports and case series.

### 4.2. Endoscopic Management of ECFs

For ECFs accessible by endoscope, advancements in endoscopic technology have provided additional options in the management of ECFs [118,119,120]. Endoscopic modalities include using stents [121], through-the-scope clips (TTSCs) [118], over-the-scope clips (OTSCs) [122,123], cardiac septal occluders [124,125,126,127], biologic mesh plugs [128], endoscopic suturing [129,130,131,132,133,134,135], tissue sealants [136], and endoscopic vacuum therapy [137].

Stents have been most frequently reported in perforations, anastomotic leaks, and fistulas affecting the upper gastrointestinal tract [138,139] (such as after esophageal [140,141,142,143,144] or gastric operations [145], including bariatric operations [121,146,147,148,149,150,151,152,153,154,155]) to cover over the internal fistula opening, preventing the influx of enzymatic fluid into the affected area, thereby giving the fistula time to heal [156]. Potential complications of stents include embedding of the stent and stent migration [138,154,157], though migration can be mitigated by suturing the stent in place [134,158,159,160]. In reported case series, the success rate of stent placement ranges from 74 to 100% [139,147,148,153,154,161,162].

The two endoscopic clip modalities currently available are TTSCs and OTSCs. For clips to be successful, the fistula orifice must be less than 3 cm in size, but recurrence remains high for this modality [163]. The OTSC overcomes some of the challenges presented by the TTSC and can be used for fistulas with an internal opening as large as 20 mm [164]. Ex vivo studies have shown that the strength of OTSC closure is approximately the same as the gold standard hand surgical suturing [165,166]. A large series of 47 patients with fistulas in the esophagus, stomach, small bowel, and colon reported a high initial technical success rate (89%), though recurrence was reported in 46% at a median of 39 days later [167]. Other authors have reported similarly high technical success rates, but higher overall success rates [168,169].

Endoluminal NPWT therapy has mainly been described in anastomotic leaks after esophageal [170,171,172,173,174,175,176], bariatric, and colorectal surgery [137,177,178,179,180], though small bowel fistula treatment has also been described [181]. Case series report high success rates ranging from 79 to 100% [162], though successful treatment requires multiple scheduled NPWT changes (2 to 3 times per week) in the operating room, up to 40 sessions [137], and sometimes requires over a month for successful healing. The most common adverse events include sponge dislocation, bleeding, and stenosis/strictures.

Although initially approved as a hemostatic adjunct, fibrin sealant has been successfully used off-label to reduce the time to fistula closure [117]. Mechanistically, it has been suggested that in addition to acting as a simple plug, the fibrin promotes ingrowth of fibroblastic tissue with eventual replacement by connective tissue. Animal studies using a controlled anastomotic leak model show very encouraging results with the application of fibrin glue [182]. Even with high-output fistulas, authors have reported success with percutaneous fibrin injection, though dilution of the thrombin component has been suggested to slow the speed of solidification to allow injection of the entire tract and cavity [183,184]. Multiple authors have reported successful case series with both endoscopic [185,186,187,188,189,190,191] and percutaneous fibrin injection to cure fistulas without requiring surgical intervention. A single-center study reported a large series of 64 patients comprising both upper and lower GI tract anastomotic leaks; these authors reported a clinical and technical success rate > 95% [192], though they cautioned that repeated sessions and large volumes of sealant are sometimes required.

The role of endoscopic suturing in fistula management continues to evolve. The potential advantages include fewer size limitations in regards to the fistula orifice, but it requires advanced technical expertise.

Multiple authors have reported success in combining the above treatments: endoVAC with fibrin glue injections [177], stents with fibrin glue [193], biologic plug with stent [128], Vicryl plug with fibrin glue [136,187,194], OTSC with stents and fibrin glue [164], and stent-over-sponge (SOS) [195].

### 4.3. Surgical Intervention

If no spontaneous closure occurs by 12 weeks following patient stabilization and optimization of nutrition, planning for definitive surgical repair should be considered [8]. Many high-volume ECF centers advocate delaying definitive reconstruction for approximately 6–12 months to allow nutritional optimization and maturation of adhesions, while emerging data suggest that earlier intervention may be feasible in selected patients [18,28]. While older studies reported that early operation is associated with twice the risk of mortality [196], a recent study reported that early operation (defined as occurring within 4 months of the last abdominal procedure) was associated with equivalent operative times and success rates compared to late operation [197]. Timing to definitive operation should be determined on a case-by-case basis.

Regardless of the timing, it can safely be anticipated that the operation will usually be long and tedious. ECF patients have usually had multiple prior operations and peritoneal contamination and extensive and dense adhesions are generally the rule. The overall steps for the operation are: (1) gain entry to the peritoneal cavity; (2) isolate the involved segment(s) of intestine; (3) resect the fistula; (4) restore GI continuity; and (5) close the abdomen. The fistulizing segment of bowel should be resected rather than primarily repaired [28]. The authors routinely employ indocyanine green (ICG) angiography to assess anastomotic perfusion, although evidence specific to ECF reconstruction remains limited [198]. Buttressing the new anastomosis with omentum (if available) may decrease the risk of recurrence [33]. Skin that surrounded the fistula or excessively thin skin should be excised due to its poor blood supply.

Abdominal wall closure may be difficult, particularly in patients with multiple prior laparotomies. Complex abdominal wall reconstruction such as anterior component separation, posterior component separation, or transverse abdominis release (TAR) with mesh reinforcement may be required for adequate and tension-free closure [199,200,201]. However, it is the authors’ experience that component separation is often difficult or impossible due to scarring from prior operations, ostomies, and drains. Sometimes, primary fascial approximation is impossible and the surgeon must place a bridging mesh and accept a planned ventral hernia. ECF patients are usually left with a reduced amount of bowel after restorative surgery, so evisceration is less of a concern in these cases.

Guidance regarding mesh type remains lacking and options include biologic, biosynthetic, non-absorbable synthetic, and absorbable synthetic materials. Each have their unique strengths and weaknesses, with biologic and absorbable options trending toward high hernia recurrence, but non-absorbable synthetic materials represent a source of ongoing foreign body infection [202]. There are accumulating data on the safety of non-absorbable mesh in the contaminated field but this has yet to be uniformly adopted [203,204]. Regardless of mesh type, every attempt should be made to achieve skin coverage. Excessive tension on the skin closure can result in ischemia with wound breakdown, so retention sutures are sometimes necessary to ensure adequate healing.

## 5. Outcomes

After definitive resection and reconstruction, the patient should be counseled to expect a prolonged postoperative recovery. Due to the extent of dissection and prolonged operative times, postoperative ileus is common and PN is often required for weeks after surgery. Pain is significant and patients usually require aggressive multimodal analgesia. At the authors’ institution, multimodal analgesia commonly includes IV acetaminophen, ketorolac, and, when appropriate, lidocaine or ketamine infusions to minimize opioid exposure. If rescue opioids are required, patient-controlled analgesia is preferred, as it allows patients a degree of autonomy and also has been shown to improve pain control and patient satisfaction [205].

Most studies place the risk of recurrence between 7 and 20% and the mortality rate between 3 and 20% [10,28,206,207]. A 2022 meta-analysis examined 53 studies published between 1975 and 2020 and included 3078 patients between the ages of 16 and 87. The recurrence rate after initial successful treatment was 11% and mortality rate was 9%. Recurrence rate was higher in patients who underwent ECF takedown in addition to abdominal wall reconstruction [12].

## 6. Future Directions

Despite significant advances in nutritional support and optimization, sepsis control, wound management, and surgical interventions, outcomes in patients with ECFs remain variable. The lack of standardized protocols regarding nutrition protocols and timing of surgical intervention has led to varying results between centers. Additionally, the presence of different underlying pathologies makes comparison in treatment protocols and outcomes variable and inconsistent. Comorbidities associated with ECFs and IF have also not been adequately explored. Many patients with ECFs and IF suffer from a variety of medical and psychosocial conditions that complicate management of this disease.

Exciting developments in stem cell applications [208] and bioengineering and materials sciences [209] hold promise for non-operative treatments, and ongoing microbiome research [210,211,212,213] suggests that preoperative dietary manipulation [214,215,216] may reduce the risk of anastomotic leaks and prevent fistulas altogether. These approaches remain investigational and currently lack sufficient evidence for routine clinical use.

## 7. Conclusions

Enterocutaneous and enteroatmospheric fistulas remain among the most challenging manifestations of acquired intestinal failure. Successful management requires more than definitive fistula closure and depends on coordinated multidisciplinary care focused on sepsis control, wound management, preservation of intestinal function, correction of fluid and electrolyte abnormalities, and optimization of nutritional status. Nutritional rehabilitation, including appropriate use of oral nutrition, enteral nutrition, parenteral nutrition, fistuloclysis, and chyme reinfusion when feasible, plays a central role in improving outcomes and preparing patients for successful definitive reconstruction.

Over the past two decades, advances in nutrition support, intestinal rehabilitation, negative pressure wound therapy, interventional radiology, and therapeutic endoscopy have substantially improved outcomes for patients with ECFs and EAFs. Future progress will likely come from further refinement of multidisciplinary care pathways, better identification of patients who will benefit from advanced intestinal rehabilitation strategies, and continued investigation of emerging therapies such as GLP-2 analogues, microbiome-directed interventions, regenerative therapies, and minimally invasive closure techniques. However, many of these approaches remain investigational and require further study before widespread adoption.

## Data Availability

All references and data analyzed and reviewed are available under References.

## Abbreviations

The following abbreviations are used in this manuscript:

ECF	Enterocutaneous fistula
EAF	Enteroatmospheric fistula
PICS	Persistent inflammation–immunosuppression catabolic syndrome
LOS	Length of stay
IF	Intestinal failure
ESPEN	European Society for Clinical Nutrition and Metabolism
IBD	Inflammatory bowel disease
IR	Interventional radiology/radiologist
NPWT	Negative pressure wound therapy
VAC	Vacuum-assisted closure
ASPEN	American Society for Parenteral and Enteral Nutrition
FELANPE	Federacion Latinoamericana de Terapia Nutricional, Nutricion Clinica y Metabolismo
EN	Enteral nutrition
PN	Parenteral nutrition
AAT	Acetaminophen absorption test
GI	Gastrointestinal
REE	Resting energy expenditure
TPN	Total parenteral nutrition
NPO	Nil per os
IFALD	Intestinal failure-associated liver disease
PNALD	Parenteral nutrition-associated liver disease
GLP	Glucagon-like peptide
SBS	Short bowel syndrome
NCBA	N-butyl cyanoacrylate
TTSC	Through-the-scope clips
OTSC	Over-the-scope clips
ICG	Indocyanine green
TAR	Transversus abdominis release
